# Evaluating Pre-Analytical Variables for Saliva Cell-Free DNA Liquid Biopsy

**DOI:** 10.3390/diagnostics13101665

**Published:** 2023-05-09

**Authors:** Blake Salfer, Daisy Havo, Skyllar Kuppinger, David T. W. Wong, Feng Li, Liying Zhang

**Affiliations:** 1 Department of Pathology and Laboratory Medicine, David Geffen School of Medicine, University of California, Los Angeles (UCLA), Los Angeles, CA 90095, USA; 2 School of Dentistry, University of California, Los Angeles (UCLA), Los Angeles, CA 90095, USA

**Keywords:** neoplasms, cell-free nucleic acids, pre-analytical phase, biomarkers, body fluids, circulating tumor DNA

## Abstract

Liquid biopsy utilizing cell-free DNA (cfDNA) has become an emergent field of study for cancer screening and monitoring. While blood-based liquid biopsy has been investigated extensively, there are advantages to using other body fluids. Using saliva is noninvasive, repeatable, and it may be enriched with cfDNA from certain cancer types. However, the lack of standardization in the pre-analytical phase of saliva-based testing is a concern. In this study, we evaluated pre-analytical variables that impact cfDNA stability in saliva specimens. Using saliva from healthy individuals, we tested different collection devices and preservatives and their effects on saliva cfDNA recovery and stability. Novosanis’s UAS preservative helped stabilize cfDNA at room temperature for up to one week. Our study provides useful information for further improvements in saliva collection devices and preservatives.

## 1. Introduction

In the field of cancer research, liquid biopsy has emerged as a less invasive technique for detailed analysis of cancer progression and diagnosis of various cancers [1]. While liquid biopsy usually refers to using blood as a biomarker source [2], saliva also offers potential in this regard as it contains disease biomarkers from the circulation, in addition to material shed directly from tumors [3]. Saliva is almost entirely composed of water; however, enzymes, hormones, proteins, cellular components, inorganic molecules, microorganisms, and food debris make up the remaining 1% of saliva [3,4,5]. Additionally, saliva contains nucleic acids such as cell-free DNA (cfDNA) and RNAs, which can be differentially analyzed to screen for cancer [5]. These nucleic acids are released by cells via apoptosis and necrosis into circulation [4]. Because the salivary glands are surrounded by capillaries, passive diffusion allows for transport of biomolecules such as cfDNA into the saliva [6]. Using salivary cfDNA from cancer patients, researchers can search for specific mutations present in the circulating tumor DNA (ctDNA) [7]. For example, Li et al. recently detected non-small cell lung cancer (NSCLC) mutations in saliva samples from lung cancer patients. They also discovered ultrashort cfDNA (uscfDNA) that is 40–60 bp long, which warrants further exploration of saliva cfDNA and the role of these short fragments [8]. Additionally, genetic sequencing analysis of the ctDNA can provide information regarding tumor evolution and heterogeneity [9]. Thus, saliva has shown promise in accurately representing the physiological state of patient’s bodies.

There are many additional reasons saliva is an appealing source for clinical use. Its collection is non-invasive and cost-efficient, and the specimen can be collected in relatively large quantities from the patient’s home [2]. Many cancer patients cannot safely donate substantial amounts of blood [10], whereas this is not an issue for saliva collection. The ability to obtain many longitudinal samples without needing trained medical personnel is yet another advantage of using salivary biomarkers to monitor cancer progression [11]. Additionally, there is some risk of infection via needle injection when collecting blood specimens [7], albeit low. Furthermore, previous studies have suggested that saliva is more enriched with tumor-derived cfDNA compared to blood plasma with respect to certain cancers such as head and neck squamous cell carcinomas [2,12,13]. However, using saliva as a cancer biomarker is not limited to cancers in close proximity to the mouth. For example, salivary transcriptome profiles have shown promise for detecting resectable pancreatic cancer with high specificity and sensitivity, and as previously mentioned, Li et al. detected NSCLC mutations [3,8]. All of these advantages make saliva an appealing source for biomarker discovery and monitoring.

Despite the many advantages and its promise in oncological research, liquid biopsy is progressing toward clinical use at a sub-optimal rate, mainly due to pre-analytical challenges [14]. For example, without a proper stabilizer, cfDNA will degrade in storage, and the blood sample may also become contaminated with cellular genomic DNA from peripheral blood cell lysis [2]. Even with proper stabilization, the processing of blood samples is required in a timely manner, usually within 6 h when collected in EDTA tubes [15]. However, alternatives such as the Streck tube and Roche tube have been shown to maintain cfDNA stability for 14 days and 7 days at room temperature, respectively [16]. Saliva lacks research regarding preservatives [17], and to our knowledge there is no commercially available saliva collector including a specific cfDNA stabilizing preservative. Additionally, cfDNA can be damaged as a result of inadequate pre-analytical handling and storage of specimens, which can result in modified nucleosides, creating falsely categorized mutations [10]. Another issue regarding sample storage is the already relatively low starting concentration of cfDNA in saliva [18]. Luckily, limitations such as low analyte volume have been overcome by new highly sensitive technologies [11], such as digital droplet PCR (ddPCR), but maximizing detectable cfDNA should still be prioritized. Thus, the low concentration of cfDNA and high susceptibility to degradation illustrates the need for an effective preservation method to maintain the quality of the sample during storage and transportation. Moreover, most liquid biopsy pre-analytical factor research has focused on blood samples. The varying biological conditions of each specimen type necessitate specific pre-analytical workflows, requiring the further study of pre-analytical factors for non-blood-based liquid biopsy and saliva specifically. By studying various biofluids, many predictive biomarkers may be discovered and thus integrated into clinical practice. The purpose of this article is to evaluate methods of storage and preservation for saliva specimens, intending to discover a preservation method that allows saliva samples to be stored for a realistic period of time without significant genomic DNA contamination or cfDNA degradation. In this study, the EGFR allele was selected as an endogenous cfDNA target for investigation due to our interest in the usage of saliva to detect NSCLC. The selected assay for EGFR exon20 covered the region of wildtype T790 as previously described [19]. Our study examined the effects of different preservatives and collection devices on the stability of cfDNA in saliva samples at various time points.

## 2. Materials and Methods

For each experiment, samples were collected from three to four healthy individuals in 50 mL Falcon tubes. The collection tubes were chilled on ice for at least 10 min prior to collection. Participants refrained from eating or drinking anything except water one hour before collection. Saliva samples (27~30 mL in total) were then pooled together into a new 50 mL tube and vortexed for 10–15 s to make a homogenous solution. Triplicate aliquots of 800 μL were prepared from the pooled sample to study the different conditions. Preservatives were added accordingly to the ratio of interest being studied. Samples were then centrifuged at 2600× *g* for 15 min at 4 °C to deplete the cells [20]. Supernatant in the amount of 0.5 mL was used, and appropriate quantities of supernatant were calculated and utilized accounting for dilution of supernatant with preservatives. This collection process constitutes our standard operating procedure (SOP).

For the saliva stability experiments, cfDNA extraction was performed immediately following collection at 1, 4, and 7 days after collection using the Qiagen Circulating Nucleic Acid Kit (Catalog #55114, Germantown, MD, USA), following the circulating microRNA protocol. The addition of carrier RNA to enhance the recovery of short cfDNA fragments was the only modification to the microRNA protocol. This protocol has been shown to recover a greater quantity of ultrashort cfDNA fragments in plasma, which were of interest to us in the saliva [21]. Samples were stored at room temperature (RT) until extraction, and extracted cfDNA was stored at −20 °C.

Droplet digital PCR was performed using 4 µL of extracted cfDNA on a QX200 droplet digital PCR system (Bio-Rad, Hercules, CA, USA). Results were reported as copies of EGFR allele per mL of saliva. All ddPCR reagents were ordered from Bio-Rad. Primers and probes were ordered from Integrated DNA Technologies (Coralville, IA, USA) and Thermo Fisher Scientific (Waltham, MA, USA), respectively. Primer/probe sequences are: Forward: 5′-GCCTGCTGGGCATCTG-3′; Reverse: 5′-TCTTTGTGTTCCCGGACATAGTC-3′; Reference probe (Vic): 5′-VIC-ATGAGCTGCGTGATGAG-MGB-NFQ-3′. Each sample was analyzed using the QX200 digital droplet reader. Data analysis was performed using the Quantasoft V1.7 analysis software.

The Student’s *t*-test was used to evaluate the performance of different collection devices and preservatives. A *p*-value < 0.05 was considered statistically significant. All figures were generated using Prism (9.5.1 Version).

## 3. Results

### 3.1. Evaluation of Collection Devices

**Figure 1:**
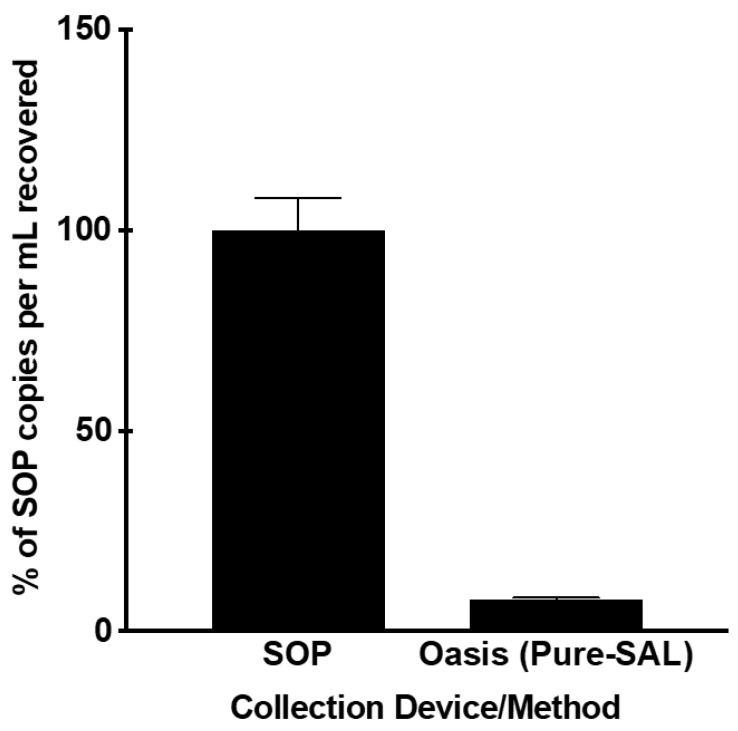
Comparison in the detection of EGFR cfDNA copies per mL of saliva with our laboratories SOP collection method and Oasis’s Pure-SAL saliva collection device. Pooled saliva samples were centrifuged immediately at 2600× *g* for 15 min at 4 °C prior to cfDNA extraction using the Qiagen Circulating Nucleic Acid Kit. Quantification of cfDNA was performed using ddPCR targeting the EGFR exon20 allele.

There are two major factors that contribute to the amount of cfDNA in the saliva specimen at the time of measurement: (1) the stability of cfDNA, i.e., whether it will be degraded when stored in the collection device; (2) whether the cells within the specimen will release DNA into the saliva specimen, which will increase the amount of cfDNA in the saliva specimen. To minimize the impacts of cfDNA released from cells within the saliva specimens, we first tested the methods that can effectively deplete cells from the saliva specimen by centrifugation following saliva collection. A ddPCR assay measuring the copies of EGFR gene was used to evaluate the impact of the collection device on saliva cfDNA recovery. We performed a head-to-head comparison of the Oasis Pure-SAL (Oasis Diagnostics, Vancouver, WA, USA) collector and our laboratories SOP collection method [22]. The Oasis device recovered 8% of the total cfDNA copies per mL of saliva compared to using the SOP collection method (*p* < 0.001) at Day 0 (Figure 1).

We also evaluated another device, the Spectrum SDNA-1000 (Spectrum Solutions, Draper, UT, USA); however, the included preservative was known to contain components that would lyse cells, introducing genomic contamination. Of the collection devices and methods tested, our researched SOP method recovered the greatest quantity of cfDNA.

### 3.2. The Effects of EDTA on Saliva cfDNA Stability

**Figure 2:**
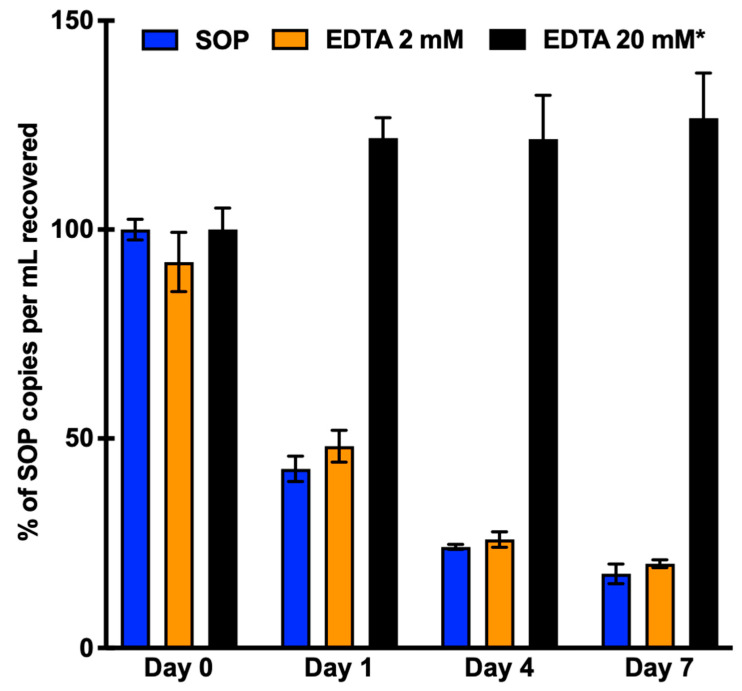
Evaluation of salivary cfDNA stability with different concentrations of EDTA at RT over the course of one week. Pooled saliva samples were centrifuged immediately at 2600× *g* for 15 min at 4 °C. Cell-free DNA extraction was performed 0, 1, 4, and 7 days after saliva collection using the Qiagen Circulating Nucleic Acid Kit. Quantification of cfDNA was performed using ddPCR targeting the EGFR exon20 allele. * A different set of pooled saliva was used for the EDTA 20 mM condition than the SOP and EDTA 2 mM condition.

As our SOP saliva collection method was able to recover the highest yield of cfDNA, we then evaluated the stability of cfDNA at room temperature over the course of a week with the addition of ethylenediaminetetraacetic acid (EDTA). EDTA is a common preservative used during blood liquid biopsy clinical testing that serves as an anti-coagulant and inhibits DNase enzyme activity. To determine EDTA’s compatibility for saliva liquid biopsy, we first tested the SOP saliva collection method with and without the addition of EDTA. In this experiment all the pooled saliva underwent centrifugation following collection to remove the cellular contents. Without the addition of EDTA, the detectable quantity of cfDNA copies per mL of saliva decreased to 43% and 18% after Day 1 and Day 7, respectively (Figure 2, blue bars). With the addition of 2 mM EDTA, the detectable cfDNA was reduced to 52% and 22% on Day 1 and Day 7, respectively, compared with Day 0. There was no statistically significant difference between the recovery of cfDNA with or without the addition of 2 mM EDTA. Thus, at the 2 mM concentration, EDTA did not significantly improve cfDNA stability (Figure 2, orange bars). As we observed a minor stability increase after 1, 4, and 7 days in the presence of 2 mM EDTA, we then tested whether a higher concentration of EDTA could improve cfDNA stability. At an EDTA concentration of 20 mM, the detectable quantity of cfDNA copies increased by 21.9% on Day 1 and 26.6% by Day 7 (Figure 2, black bars). These increases were not statistically significant (*p*-values: 0.153 and 0.238 respectively) when compared to Day 0 with the addition of 20 mM EDTA. These results confirmed our hypothesis and showed that adding EDTA at a 20 mM concentration could successfully stabilize cfDNA for one week after removing the salivary cellular components immediately after specimen collection.

**Figure 3:**
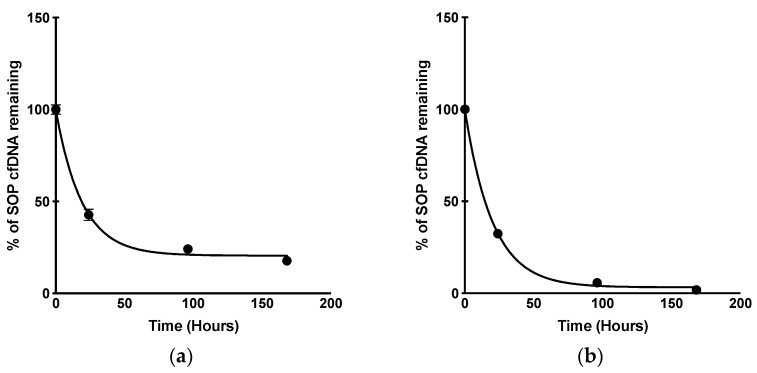
Saliva cfDNA half-life curves of unpreserved saliva collected and processed using our SOP method. (**a**), Saliva supernatant cfDNA half-life = 13.18 h. Pooled saliva samples were centrifuged immediately at 2600× *g* for 15 min at 4 °C. Cell-free DNA extraction was performed 0, 1, 4, and 7 days after saliva collection using the Qiagen Circulating Nucleic Acid Kit. Quantification of cfDNA was performed using ddPCR targeting the EGFR exon20 allele; (**b**), Whole saliva cfDNA half-life = 13.94 h. Pooled saliva samples underwent centrifugation on Days 0, 1, 4, and 7 at 2600× *g* for 15 min at 4 °C. Cell-free DNA extraction was performed following centrifugation using the Qiagen Circulating Nucleic Acid Kit. Quantification of cfDNA was performed using ddPCR targeting the EGFR exon20 allele.

Using the unpreserved SOP condition data, we were able to calculate the saliva supernatant cfDNA half-life. We first imported our data to Prism (9.5.1 Version) and used the one phase decay analysis to create a half-life curve. From this analysis, we observed a saliva supernatant cfDNA half-life of 13.18 h (Figure 3a).

### 3.3. The Effects of Delayed Processing on Whole Saliva cfDNA Stability

**Figure 4:**
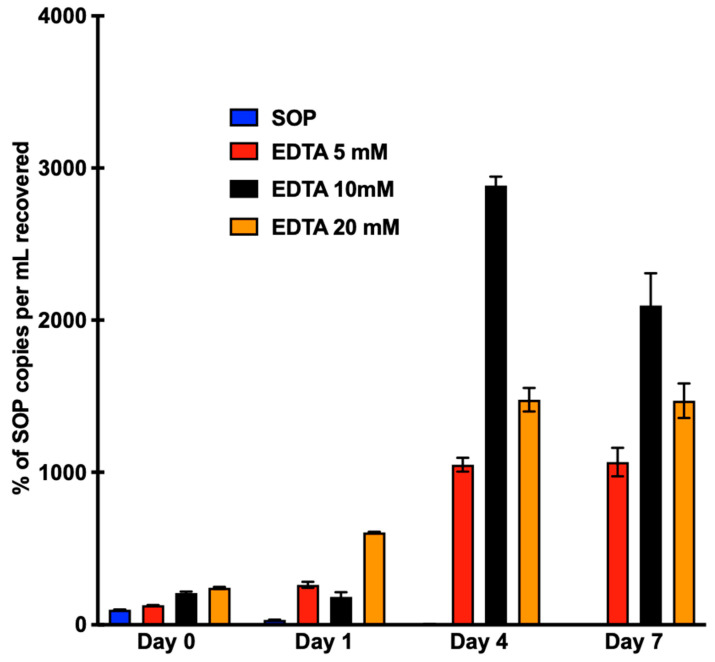
Saliva EGFR cfDNA stability at RT over the course of a week; SOP whole saliva cfDNA stability without preservative and EDTA (5, 10, 20 mM) whole saliva cfDNA stability. Pooled saliva samples underwent centrifugation on Days 0, 1, 4, and 7 at 2600× *g* for 15 min at 4 °C. Cell-free DNA extraction was performed following centrifugation using the Qiagen Circulating Nucleic Acid Kit. Quantification of cfDNA was performed using ddPCR targeting the EGFR exon20 allele.

In the previous experiments, we processed and centrifuged the saliva specimens immediately after collection. However, immediate processing may not be feasible in real practice due to the fact that many clinics may not possess the centrifugation equipment necessary for processing. We therefore designed experiments to mimic delays in processing. Such delays may be the time necessary to ship the sample from the hospital/clinic to the lab, shipping from the patient’s home to the lab, or an inability to process samples on weekends/holidays. Thus, we tested the whole saliva cfDNA stability over the course of a week at RT by centrifuging the specimens right before isolating cfDNA from the saliva at several time points, instead of centrifuging them right after collection. Similar to our saliva supernatant cfDNA stability results for the SOP condition, the detectable quantity of cfDNA drastically decreased, and in this case, even quicker, to 32% on Day 1 and 2% on Day 7 (Figure 4). Because of the scarcity of circulating tumor DNA (ctDNA) fragments in body fluids, this rapid degradation would make it extremely difficult to perform accurate mutational analysis if sample processing occurs more than 24 h after sample collection.

Like our previous experiment, from the unpreserved SOP condition data, we were able to calculate the whole saliva cfDNA half-life. The same methods and analysis were performed as previously described. From this analysis, we observed a whole saliva cfDNA half-life of 13.94 h (Figure 3b).

Aiming to increase the cfDNA stability, we tried adding three different concentrations of EDTA to the saliva: 5 mM, 10 mM, and 20 mM. At these concentrations, after four days there was a drastic increase in the EGFR copy numbers per mL, over 8-fold for 5 mM, almost 14-fold for 10 mM, and 6-fold for 20 mM (Figure 4). These results indicate a possible lytic yet preserving mechanism of EDTA that lyses the cells and preserves the released DNA. Nonetheless, this dramatic increase resulted in the search of alternative preservatives that better stabilize the cells while additionally preventing cfDNA degradation.

**Figure 5:**
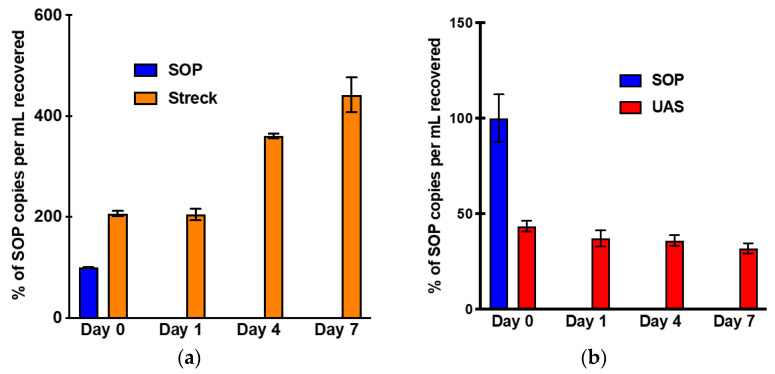
Endogenous EGFR exon20 saliva stability at RT for 1 week testing two different preservatives. Pooled saliva samples underwent centrifugation on Days 0, 1, 4, and 7 at 2600× *g* for 15 min at 4 °C. Cell-free DNA extraction was performed following centrifugation using the Qiagen Circulating Nucleic Acid Kit. Quantification of cfDNA was performed using ddPCR targeting the EGFR exon20 allele. (**a**), The number of cfDNA copies per mL of saliva recovered over the course of one week using the SOP collection method with the addition of Streck Urine Preserve at a 1:10 ratio; (**b**), The number of cfDNA copies per mL of saliva recovered over the course of one week using the SOP collection method with the addition of UAS at a 2:5 ratio.

During this search we found two preservatives, Streck Urine Preserve (Streck, La Vista, NE, USA) and UAS (Novosanis, Wijnegem, Belgium), which claimed to preserve urine cfDNA without lysing the cells. The Streck Urine Preserve was tested at a 1:10 ratio (preservative: saliva) based on Streck’s previous data [23]. The UAS urine preservative was tested at the recommended 2:5 ratio. Over the course of a week, saliva with the addition of Streck resulted in a two-fold increase between Day 0 and Day 7 (Figure 5a). This increase was concerning and indicated Streck Urine Preserve may not be the best for preserving cfDNA in saliva specimens.

Next, we tested the UAS preservative to see whether it can stabilize cfDNA over one week. While there was an initial drop in copies per mL to 43% compared to the SOP condition, there was not a statistically significant difference in copies between Day 0 and Day 7 (Red bars, *p*-value: 0.0871) (Figure 5b). Our data demonstrated that the UAS preservative was able to stabilize cfDNA for one week compared to the unpreserved saliva without extensive cellular lysis and genomic DNA contamination. For saliva specimen collection we recommend using a 50 mL Falcon tube with a pre-filled quantity of the UAS chemistry that will equate to a 2:5 preservative to saliva ratio.

## 4. Discussion

While saliva has shown evidence of harboring many relevant cancer biomarkers, pre-analytical variables such as collection devices, storage conditions, and sample processing times can greatly impact the utility of novel and existing biomarkers. In order to accurately assess the role of cfDNA biomarkers, pre-analytical standards must be established that maximize cfDNA recovery and minimize cfDNA degradation. From our experimentation, the use of the Oasis Pure-SAL collection device demonstrated insufficient recovery of saliva cfDNA. The reduction in recovery compared to the SOP condition may be due to the device’s filter, which is meant to filter the salivary cellular components. Nonetheless the limited recovery of cfDNA would make it difficult to perform ctDNA mutational analysis, especially for mutations presenting with low mutant allele frequencies.

Additionally, 20 mM EDTA was shown to stabilize the saliva supernatant cfDNA for up to one week when saliva specimens were processed immediately after collection. However, extracting cfDNA from whole saliva in the clinic immediately after centrifugation is impractical, making it difficult to take advantage of this stabilization effect. Thus, we explored adding different preservatives at multiple different concentrations to the whole saliva to determine cfDNA stability at room temperature. These experiments were meant to mimic processing delays, with the goal of finding a pre-analytical workflow that would allow for accurate mutation, fragment, and methylation analysis of salivary cfDNA and ctDNA within a week of collection. From our test, unpreserved saliva cfDNA degrades rapidly, and we can expect to lose over half of all detectable cfDNA within 24 h. Using time points over the course of 1 week, salivary supernatant cfDNA and whole saliva cfDNA half-lives were calculated to be 13.18 and 13.94 h, respectively. Previously, Wang et al. reported an in vitro salivary DNA half-life of 4.19 h at room temperature. In their study, saliva did not undergo centrifugation and underwent a freeze–thaw cycle at −20 °C. Genomic DNA was extracted, and DNA concentration was measured using a micro-spectrophotometer [24]. It is thus difficult to compare our results with theirs given the discrepancies in pre-analytical factors, time points, and analytical methods. However, both results support rapid degradation and timely extraction. For other bio specimen types, such as blood, half-lives of approximately 15 min to 2.5 h have been reported [25]. In urine, the reported half-life ranges from undetectable to 2.6–5.1 h [26]. The differences in the biological makeup, such as nuclease expression levels, of each liquid biopsy media make half-life analysis difficult. Further study could seek to validate cfDNA half-lives in various specimen types, as well as focusing on maximizing cfDNA stability through preservation. The latter is something we tried to address throughout our experimentation. Additionally, further studies should aim to compare matched saliva and plasma specimens from patients to determine specific roles of each biological sample in cancer progression.

To perform accurate downstream analysis the saliva cfDNA must be processed quickly or preserved. Of the commercially available preservatives, we were unable to find any saliva-specific chemistries preventing cellular lysis and cfDNA degradation. We chose to test the Streck Urine Preserve and UAS chemistries because they previously exhibited these properties in urine, and UAS had shown preliminarily promising results in saliva as well. Both EDTA, at the tested concentrations, and the Streck Urine Preserve resulted in an increase in cfDNA recovery after 7 days, likely due to cellular lysis and genomic DNA contamination. This contamination would make it harder to detect specific ctDNA mutations and could distort the results of further analysis of cfDNA features. Likely, the biological properties of saliva make whole saliva incompatible with these commonly used blood and urine preservatives. However, the UAS preservative was shown to be relatively stable, and there was no statistical difference between cfDNA copies recovered on Day 0 and Day 7. The initial reduction in UAS copies per mL recovered may be due to the interaction between the UAS preservative and cfDNA. We have hypothesized that some of the cfDNA fragments may be lost during centrifugation due to an interaction with UAS. Yet, the mechanism is currently unclear and warrants further study.

A saliva collection device specifically designed for cfDNA analysis ideally would include a preservative, such as UAS, that stabilizes cfDNA for a week at room temperature and is easy for the patient to use. With such a device, many pre-analytical variables could be mitigated and salivary ctDNA could become an even more reliable biomarker for cancer detection and monitoring, especially for carcinomas close to the oral cavity.

It is important to note that our study has limitations. Pooled saliva was collected from eight healthy individuals and is unlikely to completely represent a larger population. Additionally, most of these individuals were 20–30 years old and could have significantly different salivary biological properties as well as cfDNA levels compared to an older cohort. Results may vary on a patient-to-patient basis due to individual differences in saliva biology. In addition, saliva collection was not completed at a specific standardized time for each experiment. Lastly, we did not test any cancer patient samples. Patients with cancer may have different salivary cfDNA levels or the preservatives could interfere with mutation detection. In the future we hope to test cancer samples with our optimized collection and stabilization methods, possibly including the analysis of tissue-based matched samples. Other future considerations include testing of new commercial saliva collection devices and preservatives.

Setting aside the limitations, it is crucial to standardize the pre-analytical variables for saliva liquid biopsy to create more accurate, noninvasive, and easily reproducible clinical tests. Our study provides useful information for further improvements in saliva collection devices and preservatives. Additionally, it provides information on these pre-analytical factors and has paved the path for developing screening and monitoring tests for cancer detection and patient management.

## 5. Conclusions

In conclusion, of the preservatives we tested, UAS stabilized salivary cfDNA at room temperature best, in combination with our SOP collection method. A more ideal collection device and further standardized pre-analytical conditions likely exist, warranting further research.

## Data Availability

Data supporting the reported results can be obtained on request.
